# The Effect of Upper Extremity Slings (UESs) on Balance in Young Adults

**DOI:** 10.7759/cureus.42724

**Published:** 2023-07-31

**Authors:** Ben S Rhee, Neel Vishwanath, Soryan Kumar, Loree K Kalliainen

**Affiliations:** 1 Medical School, The Warren Alpert Medical School of Brown University, Providence, USA; 2 Plastic Surgery, The Warren Alpert Medical School of Brown University, Providence, USA

**Keywords:** conservative rehabilitation, postural balance, upper extremity rehabilitation, balance impairment, sling

## Abstract

Introduction: Upper extremity slings (UESs) are frequently provided for patients with a hand or forearm injury. However, their effect on balance has not been well explored. We sought to characterize the effect of a UES on balance in young adults.

Methods: Healthy young adult participants with no injuries acting as a proxy for the general young adult patient population using UESs balanced on a BioDex Balance System platform: once while wearing a UES and once without wearing it, to serve as their own control. Participant weight, height, gender, hand dominance, overall stability index, anterior/posterior stability index, and medial/lateral stability index were recorded. Comparisons were analyzed with paired t-tests and linear regression analysis.

Results: No significant difference in the three stability index scores were found between UES and no UES usage. Height and weight were found to have positive significant relationships with the overall stability index during UES usage.

Conclusions: Our study demonstrates the feasibility of assessing balance discrepancies between the sling and nonsling usage in a broader patient population and suggests that height and weight may impact balance negatively during UES use.

## Introduction

Upper extremity slings (UESs) are a commonly used rehabilitation tool that primarily serves as a support for the upper extremity to decrease pain, minimize unwanted motion, and improve tissue and bone healing [1]. Balance is the ability to maintain the body’s center of gravity over its base of support, and its impairment may occur due to visual, cerebellar, or vestibular dysfunction, as well as a result of proprioceptive and motor deficits [2]. Balance impairment is a serious issue because it increases the risk of falls, which can drive injury, hospitalization, and increased healthcare utilization [3-4]. The upper extremity is a major source of proprioceptive feedback to the central nervous system, integrating information for proper postural control and balance [1, 5]. Thus, sling immobilization of the upper extremity may impact proprioceptive feedback and impair balance. 

A body of research has studied this phenomenon in hemiplegic patients, who are often prescribed a UES for their affected arm to prevent shoulder subluxation. A prospective study by Lui et al. demonstrated that UES use led to significant balance decompensation as measured by computerized dynamic posturography [1]. In contrast, other literature has shown that there may be benefits to UES use in hemiplegic patients, but only in the early phase of stroke rehabilitation [6].

The majority of UESs are used following sports and trauma-related injuries or following upper extremity surgery [7-8]. Common fracture locations for which UESs are used include the acromioclavicular joint area, clavicle, proximal humeral shaft, olecranon, distal radius, wrist, and metacarpals. They can also be used in conjunction with other immobilization methods, such as splinting [9]. While studies exist quantifying the potential benefits of sling use in these patient populations (i.e., reduction of early-onset pain after fracture, increased preservation of upper extremity function), the impact on balance has not been explored [8, 10]. In addition to greater imbalance, potential downsides may include the development of stiffness, skin irritation, and interference with daily activities. Furthermore, while UESs are useful for the upper arm, their use has also been extrapolated for the forearm, wrist, and hand -- these are areas for which a sling may not be necessary and no concrete benefits are conferred. In particular, the senior surgeon has noted a pattern of falls in patients with forearm/wrist/hand injuries who are using slings. UESs may be potentially needlessly used for parts of the arm while adding the risk of falls. We, therefore, sought to (1) analyze the effect that wearing a UES has on the balance of healthy young adult subjects, as a proxy of the general young adult patient population requiring a UES, and (2) examine any associations between patient factors and balance with and without UES usage. This study was done in part as a pilot study to develop techniques and assess safety prior to generalizing the work to a broader patient population.

This article was previously presented as a poster at the Annual Scientific Meeting of the Rhode Island Chapter, American College of Physicians (RI ACP) on March 15, 2023. 

## Materials and methods

A prospective crossover study was conducted on healthy young adult participants with no active injuries. It was approved by the Lifespan IRB (Protocol #1825789) prior to participant recruitment. Participants were recruited through email listservs and public bulletin advertisements in the Providence, RI, area. Inclusion criteria were aged between 18 and 40 and no current musculoskeletal injury was reported. Respondents who did not fit these criteria were not included in the study. The participants were seen at an outpatient physical rehabilitation center between August and November, 2022. Before undergoing the trials, each participant was visually checked to confirm no ongoing injuries or recuperation from injury, as well as for no issues with gait or any other current physical limitations. During the sessions, each participant’s balance was evaluated using a BioDex Balance System platform. The BioDex Balance System machine is a balance screening and training tool that can analyze the balance competence of its users [11]. All participants were evaluated on the BioDex Balance System once while wearing a UES (on the right upper extremity only) and once without. The crossover occurred in one session, such that a participant would undergo a trial (either sling off or on), wait for 3 min, and then undergo the second trial with the other sling mode. Trials without a UES served as a control and were compared to trials during which a UES was worn. The order in which each participant balanced with or without a UES was randomized through random number generation in Microsoft Excel.

During each trial, participants were instructed to attempt to maintain their center of balance on a destabilized platform set at the machine’s lowest resistance level. This was measured by a tracking sensor and reflected on the monitor display’s cursor superimposed on a circle representing the platform that the patient could use to correct their balance deviation as the trial progressed. Each trial was implemented for 20 s, and overall stability index, anterior/posterior stability index, and medial/lateral stability index scores were recorded for each iteration. These index scores were displayed on the BioDex Balance System after each trial and serve as a quantitative measure of how well participants were able to maintain balance. They represent the variance of platform displacement in degrees from level, with a higher number indicative of the participant having more trouble balancing. Overall stability index represents the variance in foot platform displacement in degrees, from level, in all motions during a trial, while anterior/posterior and medial/lateral stability indices represent such variance for motion in the sagittal and frontal planes only, respectively [12]. 

Participant demographics (age, ethnicity, race, height, gender, and dominant hand) were recorded. Differences in patient balance with and without UES were analyzed using paired t-tests. Associations between demographic factors (height and weight) and balance index scores were assessed using linear regression, adjusted for sling use order.

## Results

All respondents who met the inclusion criteria were enrolled (25 females, 27 males). On average, participants were 23 years old, 155 pounds, and 68 inches (Table 1). All but one participant was right-handed. The average overall stability index score was 3.80. Average anterior/posterior and medial/lateral stability index scores were 2.35 and 2.12, respectively. No significant difference in the overall stability index score was found between UES usage and no UES usage (p = 0.44). There was also no significant difference in anterior/posterior stability index score between UES usage and no UES usage (p = 0.36). Similarly, no significant difference was found for medial/lateral stability index score between UES usage and no UES usage (p = 0.29).

**Table 1:** Summary of participant baseline characteristics (n=52). *Mean (SD) SD, standard deviation

Characteristic	Average value or frequency
Age	22.8 (3.8)*
Weight (lbs.)	154.9 (28.9)
Height (in.)	67.4 (3.7)
Gender	
Male	27
Female	25
Dominant Hand	
Right	51
Left	1

**Figure 1:**
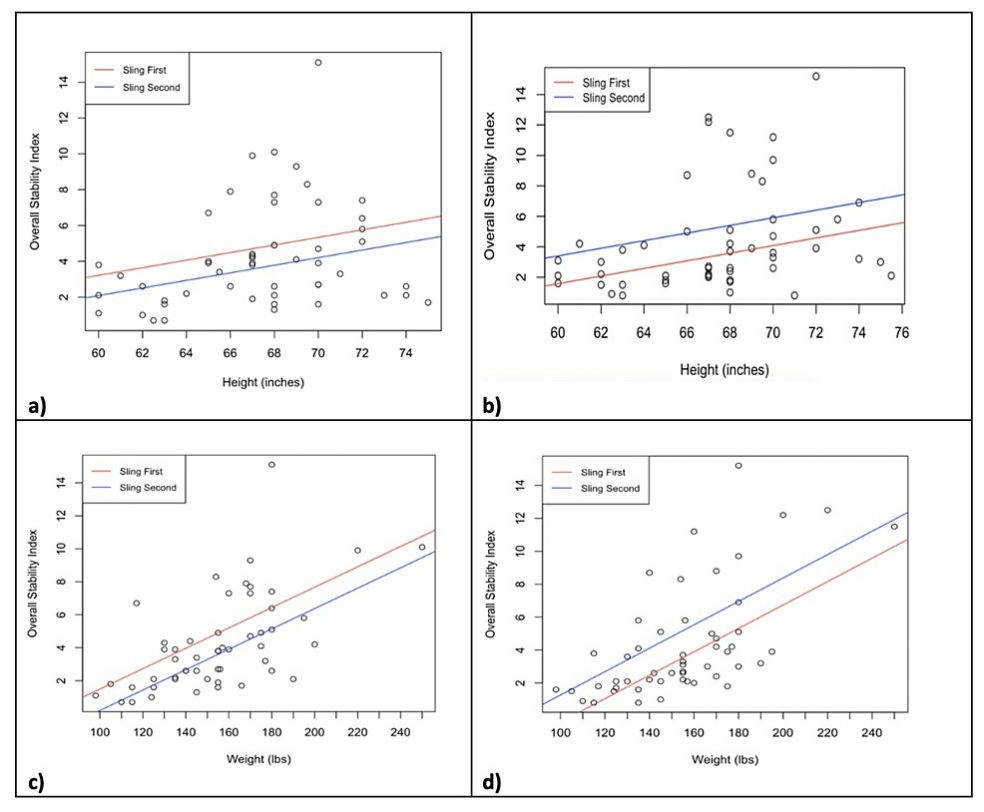
Regression lines comparing demographic factors (height, weight) and OSI scores during UES and non-UES usage, adjusted for sling order. a) Height (in.) vs. OSI during UES usage. b) Height vs. OSI during no UES usage. c) Weight (lbs.) vs. OSI during UES usage. d) Weight vs. OSI during no UES usage. A significant positive relationship was found in all comparisons. OSI, overall stability index; UES, upper extremity sling

Figure 1 shows regression lines between overall stability and demographic factors (height, weight) adjusted for sling use order during UES and no UES usage (note that each plot depicts two lines given the binary choice of sling order). During both UES and no UES usage in our cohort, increased height (inches) was significantly associated with an average increase of 0.21 (p = 0.048) and 0.25 (p = 0.033) in the overall stability index, respectively. During both UES and no UES usage, increased weight (pounds) was significantly associated with an average increase of 0.062 (p < 0.001) and 0.071 (p < 0.001) in the overall stability index, respectively.

For associations between height/weight and anterior/posterior stability, during UES usage in our cohort, increased height was not significantly associated with the anterior/posterior stability index (p = 0.058). When participants did not wear a UES, increased height was significantly associated with an average increase of 0.21 in the anterior/posterior stability index (p = 0.029). During both UES and no UES usage, increased weight was significantly associated with an average increase of 0.045 (p < 0.001) and 0.058 (p < 0.001) in the anterior/posterior stability index, respectively.

We found no significant relationship between height and medial/lateral stability index during both UES usage (p = 0.057) and no UES usage (p = 0.11). However, during both UES and no UES usage, increased weight was significantly associated with an average increase of 0.033 (p < 0.001) and 0.03 (p < 0.001) in the medial/lateral stability index, respectively.

## Discussion

In this study, we sought to characterize the effect that wearing an UES has on balance in a general young adult population by comparing stability index scores during sling and non-sling usage for healthy subjects. In turn, this data could further inform clinical decision-making of when and for how long to use a UES in otherwise healthy patients with forearm and hand injuries for whom the sling is not needed to directly stabilize an injury. Our results indicate there was no significant difference in stability between UES and non-UES usage in our cohort. There are significant inverse linear relationships between height and overall stability during UES usage, as well as between weight and overall stability during both UES and non-UES usage. 

The significant inverse linear relationships found between height/weight and overall stability are noteworthy, given that previous literature has found that increased height and body mass index can impact balance in both positive and negative ways. For instance, in their study assessing the effect of height and BMI on computer dynamic posturography (CDP) parameters in young healthy women without balance disorders, Olchowik et al. found that the CDP parameter, motor strategy (MS), was lower in taller and higher-BMI women. This meant that for those women, their ankle muscles’ activity tended to decrease as hip muscle’s activity increased while trying to maintain posture during CDP trials. However, the postural response latency (time between the onset of change in body position and muscle reflex in response to that change) increased with height in the same study, indicating that taller women were not able to reflexively respond as quickly to body position changes as their shorter counterparts could [13]. Greater postural response latency has also been documented in patients with MS compared to healthy controls, and this variable contributes to disturbances in balance control for such patients [14]. 

In their analysis of the effect of height on the test-retest reliability of center of pressure (COP) variables during postural balance performance in healthy young adults, Eom et al. found that shorter subjects exhibited smaller variation in postural sway and area compared to the taller subjects [15]. Obesity has also been found to have a negative impact on balance -- Dutil et al. demonstrated that in older women, more obese subjects tended to spend less time in stability zones while balancing on a force platform than normal-BMI subjects. This study hence concluded that obesity in older women could be considered a potential contributing factor for falling [16]. Given that many parameters are involved in the determination of postural stability, it is not surprising that certain balance variables are correlated with height/BMI in a disparate fashion. While many demographic characteristics factor into how well one can balance, it may be prudent to carefully consider the use of UES in taller, heavier patients if they are not absolutely necessary for protection and healing as postural stability may be negatively affected.

While our study did not find significant differences in stability scores stratified by gender or age, these characteristics should also be considered when assessing factors that can affect postural stability. A study measuring the effects of restricted sensory input and surface destabilization through the use of a force platform on balance in elderly community dwellers found that female participants more often lost balance during trials with eyes closed and during backward destabilization of the platform than male participants did [17]. Wearing a sling is also a condition that can stress balance capability and limit postural control, and thus may lead to greater destabilization for elderly females as was demonstrated by the aforementioned study. When stratifying by age alone, there is also evidence to suggest a greater degree of postural imbalance by such stratification. Liaw et al. demonstrated that compared to young subjects, elderly subjects had a higher degree of postural imbalance and greater use of hip strategy to maintain balance when standing on a swaying support surface in the absence of visual surround or with conflicted visual surround [18]. Once again, these findings were concluded in the setting of conditions that are designed to stress balance capability, which sling usage may also impede. 

There are limitations to our study. Our cohort was not a truly accurate representative sample of the general patient population requiring a UES. None of our study subjects had a musculoskeletal injury and were young adults. Since they did not have injuries, the balance may be less negatively affected compared to a population with an injury. Future studies will recruit older subjects recuperating from an upper extremity injury as they are at even greater risk for balance issues during sling use. We have demonstrated that this assessment can be performed safely on a young, healthy population and can now extrapolate the investigation to a more at-risk population. Future studies should also recruit a greater number of participants to increase the power of the study compared to our pilot investigation. 

## Conclusions

Overall, our pilot study demonstrates the feasibility of assessing balance discrepancies between sling and non-sling usage in a safe environment and adds to the currently sparse literature on the effects of upper extremity sling use on balance. Our study also suggests that certain demographic factors such as weight and height may negatively impact balance during UES usage in young adults. In the future, we plan to conduct a study incorporating older subjects with upper extremity injuries to further investigate the relationship between balance and sling use. 
